# New Aspects in Metabolism of Aging

**DOI:** 10.1093/geroni/igab046.1757

**Published:** 2021-12-17

**Authors:** Rozalyn Anderson

## Abstract

In recent years there has been a renewed emphasis on metabolism as a key contributor to a host of chronic non-communicable conditions: cancer, neurodegeneration, frailty, and functional declines in immune and inflammatory processes. All share a common connection in metabolic dysfunction. Furthermore, aging itself is associated with changes in metabolism, although the underlying drivers for these changes are unknown. Here we introduce speakers working at the cutting edge in metabolism research, and whose studies are of direct relevance to aging. Dr. Chandel will focus on mitochondrial biology, describing recent advances in understanding the mechanisms of the beneficial effects of metformin. Dr. Haigis takes the mitochondrial theme to cancer biology, the area of research that revived metabolic perspectives in biomedical research. Dr. Najt’s talk describes a less well studied organelle, the lipid droplet, and its role in a rapidly expanding area of research on lipid metabolic regulation specifically in the context of aging. Dr. Brown-Borg will present data on nutritional and genetic modulation of metabolism and how pathways converge to influence chromatin and epigenetic regulation of gene expression. Together our speakers explore new concepts in metabolism research that are of particular relevance to aging. This session aligns with the concept of GeroScience, the more we know of aging biology the better we understand diseases and disorders of aging. This session will demonstrate that metabolism, its regulation, and its influence on key processes linked to health and longevity, place it in a central position as we seek to discover targets and interventions to improve human aging.

